# Fibrotic Myofibroblasts Manifest Genome-Wide Derangements of Translational Control

**DOI:** 10.1371/journal.pone.0003220

**Published:** 2008-09-16

**Authors:** Ola Larsson, Deanna Diebold, Danhua Fan, Mark Peterson, Richard Seonghun Nho, Peter B. Bitterman, Craig A. Henke

**Affiliations:** 1 Pulmonary Division, Department of Medicine, University of Minnesota, Minneapolis, Minnesota, United States of America; 2 Department of Biochemistry, McGill University, Montreal, Canada; 3 School of Public Health Division of Biostatistics, University of Minnesota, Minneapolis, Minnesota, United States of America; National Heart and Lung Institute, United Kingdom

## Abstract

**Background:**

As a group, fibroproliferative disorders of the lung, liver, kidney, heart, vasculature and integument are common, progressive and refractory to therapy. They can emerge following toxic insults, but are frequently idiopathic. Their enigmatic propensity to resist therapy and progress to organ failure has focused attention on the myofibroblast–the primary effector of the fibroproliferative response. We have recently shown that aberrant beta 1 integrin signaling in fibrotic fibroblasts results in defective PTEN function, unrestrained Akt signaling and subsequent activation of the translation initiation machinery. How this pathological integrin signaling alters the gene expression pathway has not been elucidated.

**Results:**

Using a systems approach to study this question in a prototype fibrotic disease, Idiopathic Pulmonary Fibrosis (IPF); here we show organized changes in the gene expression pathway of primary lung myofibroblasts that persist for up to 9 sub-cultivations in vitro. When comparing IPF and control myofibroblasts in a 3-dimensional type I collagen matrix, more genes differed at the level of ribosome recruitment than at the level of transcript abundance, indicating pathological translational control as a major characteristic of IPF myofibroblasts. To determine the effect of matrix state on translational control, myofibroblasts were permitted to contract the matrix. Ribosome recruitment in control myofibroblasts was relatively stable. In contrast, IPF cells manifested large alterations in the ribosome recruitment pattern. Pathological studies suggest an epithelial origin for IPF myofibroblasts through the epithelial to mesenchymal transition (EMT). In accord with this, we found systems-level indications for TGF-β -driven EMT as one source of IPF myofibroblasts.

**Conclusions:**

These findings establish the power of systems level genome-wide analysis to provide mechanistic insights into fibrotic disorders such as IPF. Our data point to derangements of translational control downstream of aberrant beta 1 integrin signaling as a fundamental component of IPF pathobiology and indicates that TGF-β -driven EMT is one source for IPF myofibroblasts.

## Introduction

Fibroproliferative disorders are a major cause of morbidity and mortality [1]. Traditionally parsed into categories based on the target organ afflicted-lung, liver, kidney, heart, vasculature, CNS or integument-biomedical scientists now view the fibroproliferative diseases as sharing a common pathobiology independent of the organ or tissue that scars [2], [3]. Organs can heal or scar following toxic exposures, with fibrosis predominating when the injurious agent cannot be eradicated by the host defense system, as occurs with certain infections; or is repeatedly introduced over a protracted interval of time as occurs in asbestosis, silicosis or alcohol-induced hepatic cirrhosis. The clinical focus in these situations is specific antimicrobial therapy or prevention. More vexing, however, are the fibroproliferative diseases of unknown cause, which frequently progress to organ dysfunction or death. Currently, while there are a few therapeutic leads [4], [5], there are no therapies that reproducibly interdict fibrosis.

For decades, innate and adaptive immunity has served as the focal point for studies of tissue fibrosis. While an unremitting immune response can lead to fibrosis, in many idiopathic fibrotic disorders, immune-suppressive therapy affords limited benefit. This has shifted attention to the primary effector of the fibroproliferative response, the fibroblast itself. Fibroblasts from patients with systemic sclerosis, renal fibrosis, keloids and pulmonary fibrosis display aberrations in processes that govern nearly every aspect of the fibroproliferative response [6], [7], [8], [9], [10], [11]. These data indicate that fibrotic fibroblasts manifest pathological control of pathways governing proliferation, viability, motility, contractile function and connective tissue production. It is noteworthy that while these differences emerge in the context of exogenous signals from matrix, cytokines, chemokines, morphogens and peptide growth factors; fibrotic fibroblasts appear to retain a distinct cell biology *in vitro*.

Here we study the fundamental pathobiology of tissue fibrosis by focusing on a lethal respiratory disorder, idiopathic pulmonary fibrosis (IPF), as a prototype fibroproliferative disease. The histological pattern of IPF is usual interstitial pneumonitis (UIP), a patchy fibroproliferative process that spares some respiratory units while affecting others nearby. Progression of fibrosis leads to obliteration of the gas-exchange surface. This pathological respiratory phenotype is the culmination of complex interactions among myofibroblasts, epithelial cells, cytokines, and the surrounding extracellular matrix. Myofibroblast foci, the pathologic hallmark of IPF, are comprised of myofibroblasts embedded in a type I collagen rich matrix [12], and the burden of myofibroblast foci found in lung biopsy samples inversely correlates with patient survival [13], [14], [15]. Whereas myofibroblasts in healing wounds contract their matrix and undergo apoptosis in a timely manner, myofibroblasts in IPF lesions persist. The mechanism involves aberrant beta 1 integrin signaling in response to type I collagen. This results in defective PTEN function and unrestrained Akt signaling leading to downstream activation of the translation initiation machinery [16]. How this pathological integrin signaling alters the gene expression pathway of fibrotic myofibroblasts has not been elucidated.

To answer this question, we took a systems biology approach and examined two key steps in the myofibroblast gene expression pathway genome-wide–transcription and ribosome recruitment. Transcriptional control in IPF has been previously characterized in lung tissue samples [17]; however, ribosome recruitment pattern-a measure of which transcripts are being translated into protein-has not been examined in tissue or cell lines. We elected to carry out this analysis using primary lung myofibroblasts in 3-dimensional type I collagen gels, an *in vitro* system that surrounds myofibroblasts in type I collagen in a context that lacks exogenous cytokines. To simulate an aberrant, fibrotic environment, we studied cells in type I collagen gels that were fixed to the sides of a tissue culture dish and therefore not allowed to contract (referred to as “non-contractile” matrices); to simulate the environment of physiological healing, we released the type I collagen gels from the sides of the dish and allowed the myofibroblasts to contract their matrices (referred to as “contractile” matrices) [18]. By examining the gene expression pathway of control and IPF myofibroblasts in both non-contractile and contractile collagen gels, we are able to determine the extent to which matrix type and the tissue of origin accounts for any differences observed at two levels of gene expression regulation.

Here we show distinct intrinsic differences in the gene expression pathway between control and IPF myofibroblasts in both non-contractile and contractile type I collagen matrices. While differences are present at the transcriptional level, the majority of differences observed are at the level of ribosome recruitment. Importantly, we demonstrate that IPF myofibroblasts manifest a much greater dependence on collagen matrix conditions than do their control counterparts, changing the translational activity of a large set of transcripts. Our data indicate that IPF myofibroblasts are intrinsically pathological cells with fundamental changes in their gene expression pathway primarily at the level of ribosome recruitment regulation.

## Methods

### Cell Line Procurement and Characterization

Human primary myofibroblasts from twelve different donors were utilized (this study was approved by the University of Minnesota Institutional Review Board for Human Subjects Research). These consisted of six control samples (histologically normal lung distant from resected tumor) and six samples from patients with IPF (histologically confirmed UIP). Tissue was obtained at the time of biopsy, autopsy, lung resection or lung transplantation following procedures approved by the University of Minnesota Institutional Review Board for Human Subjects Research. Previous work performing microarray analysis on normal lung tissue[19] has suggested significant differences when comparing samples from men and women and also when comparing patients older than 60 years with patients younger than 40 years of age. Patients in our study were not significantly different in terms of gender (p = 0.43) or age (IPF range 57–68, control range 56–82, p = 0.14) at the time of tissue procurement. Lung tissue explants were cultivated in 35 mm tissue culture dishes in explant medium (DMEM+20% FBS+antibiotics and antimycotics) at 37°C in 95% air, 5% CO2. Outgrowth was evident in 5 to 7 days, and cells filled the dish in 2 to 3 weeks. Cells from each 35 mm dish were released with trypsin-EDTA and placed in 100 mm tissue culture dishes after trypsin was neutralized with fresh explant medium. These cells, designated passage 1, were cultivated in growth medium (DMEM+10% FBS+antibiotics) at 37°C in 95% air, 5%CO2. Medium was replaced twice weekly, and cells were subcultivated weekly at a 1∶4 split ratio. Cells designated myofibroblasts in both IPF and control samples had typical spindle morphology, were vimentin- and alpha smooth muscle actin-positive; and factor VIII- and surfactant C-negative. Cells used in this study were between passage 4 and 9.

### Assessment of Proliferative Uniformity

Myofibroblasts in log phase growth were released from culture dishes with Trypsin-EDTA, washed, suspended in PBS containing 2.5 µM of the stable vital dye carboxyfluoroscein succinimidyl ester (CFSE) (Sigma) and incubated at 37°C for 10 minutes (shaking every 2 minutes). The reaction was stopped with ice cold PBS. Cells were centrifuged (1000 g) and washed with PBS. The resultant CFSE labeled myofibroblasts were placed into 6 well clusters at 40,000 cells/well in growth medium (DMEM+10% FBS) and cultures continued (37°C, 5% CO2). At the time points indicated, cells were released from the culture dish and fixed (4% formaldehyde) prior to analysis by FACS (day 0 cells were harvested 4 h after seeding).

### Collagen Gel Preparation

Collagen was obtained from Cohesion Corporation, Palo Alto, CA. Cells were removed from tissue culture plates using trypsin and mixed with DMEM, 10% FBS and collagen (final collagen concentration 0.5 mg/ml). This mixture was polymerized in a water bath at 37° C, aliquoted into 3.5 cm tissue culture dishes, and placed into an incubator at 37° until harvest. Final cell density was approximately 200,000 cells/ml. Tissue culture plates for non-contractile gels had been pre-coated with collagen 100 µg/ml in phosphate-buffered saline (PBS) in order to prevent matrix contraction. Cells in these non-contractile collagen matrices were incubated at 37°C for 6 hours. Contractile matrices were prepared as above; they were allowed to polymerize in uncoated tissue culture dishes for two hours, and then the gel was released by tapping the side of the dish. They were placed in an incubator for four more hours before harvesting (for a total 6-hour incubation time, equal to non-contractile matrices). Degree of collagen matrix contraction was not significantly different between IPF and control myofibroblasts (data not shown).

### Polyribosome Preparation

Cells were harvested in log phase using trypsin and incorporated into collagen gels as described above. Myofibroblasts were collected from the gels at the predetermined time point using collagenase 5 mg/ml (Sigma, St. Louis, MO) containing cycloheximide (100 µg/ml) and collected by centrifugation. A small portion of non-homogenized cells was retained for Trireagent (Sigma) processing to isolate total cellular RNA (designated “total RNA”) for microarray analysis. The remaining cells were used for polyribosome preparations as described [20]. Ten 0.5 ml fractions were collected from each sample into tubes containing 50 µl of 10% SDS. RNA from each fraction was processed using Trireagent according to the manufacturer's directions and precipitated with isopropanol. Fractions 7–10, consisting of mRNA with four or more bound ribosomes, designated “heavy”, were pooled for microarray analysis.

### Microarray Hybridization

Starting with 10 µg of ribosome-bound or total RNA, conversion to labeled cRNA was performed using the One Cycle Target Labeling and Control Reagent Kit according to the manufacturer's directions (Affymetrix Corp., Santa Clara, CA). Labeled fragmented cRNA (20 µg) was submitted to the University of Minnesota Biomedical Genomics Center and probed with Affymetrix U133plus2 microarrays.

### Quantification of mRNA by Real Time PCR

A new set of polyribosome RNA preparations, different from those used to perform the microarray analysis, was used for real time PCR. We chose one IPF and one control myofibroblast primary cell type for this set of experiments. RNA from each fraction of the sucrose gradient was extracted using Trireagent and quantified. An internal standard, “alien RNA”, was spiked into each sample to control for differences in cDNA conversion efficiency as described in the instructions for the Alien® QRT-PCR Inhibitor Alert kit (Stratagene, LaJolla, CA). cDNA was synthesized from 2.0 µg of each fraction using Taqman Reverse Transcriptase Reagent Kit (Applied Biosystems, Foster City, CA) primed with oligo dT. Primer sequences for selected genes were selected using the DNASTAR program (DNASTAR, Inc., Madison, WI), and the resulting sequences were synthesized in the University of Minnesota microchemical facility. Real time PCR was performed using a LightCycler FastStart DNA Master^PLUS^ SYBR Green I Kit (Roche Diagnostics, Indianapolis, IN). 2.5 ul of the cDNA product was used for amplification of each sample. Primer sequences were as follows: CFL2 forward 5′GGA CCG TTC GAC ACT TGG AGA3′ CFL2 reverse 5′AAT GGA CTG AGC TGG AGA AAT GG3′; PDCD8 forward 5′CAG CGA TGG CAT GTT CCT CTA3; PDCD8 reverse 5′ACG CGG CCT TTT TCT GTT TCT3′; FUT10 forward 5′AGC AGC GCG AGA GTA GAA GTG AAT3′; FUT10 reverse 5′CAG TAG ATG CCC CAG ACA GGA GAG3′. Samples were quantified at the log-linear portion of the curve using LightCycler analysis software and compared to an external calibration standard curve. Each sample was normalized for cDNA conversion efficiency using the external “alien control”. The total RNA samples were normalized using β-actin. β-actin primer sequences were: forward 5′ CTG GAA CGG TGA AGG TGA CA 3′, reverse 5′ AAG GGA CTT CCT GTA ACA ATG CA 3′.

### Western blotting

Cells were grown to 70% confluence and serum starved for 48 hr. Cells were released with trypsin and seeded onto 100 mm dishes that were pre-coated for 1 hr with Pur-Col monomeric collagen 100 ug/ml in PBS (Allergan Sales Inc.). Cells were allowed to adhere for 75 min, mechanically released and resuspended in lysis buffer (40 mM tris pH 7.5, 300 mM NaCl, 2 mM EDTA, 100 mM NaF, 2% NP-40, 1% Na Deoxycholate, supplemented with “Complete” protease inhibitor tablets (Roche)). Lysates were kept on ice for 10 min, centrifuged at 12,000G for 10 min with supernatants retained and subjected to electrophoresis and Western blotting with antibodies for Keratin 18, PDCD8 and CFL2 from Cell signaling (Boston, USA); FUT10 from Abcam (Cambridge, USA); and β-actin from Sigma (USA).

### Data analysis

Totally 12 samples (6 IPF and 6 controls) informed the study. From each sample we obtained the polyribosome bound (4 or more bound ribosomes) and total RNA under the two conditions under study, thus totally 48 hybridizations. The data was normalized using GCRMA and updated probe sets definitions “RefSeq v7” as defined in [21] as these provide improved precision and accuracy [22]. We used the Significance Analysis of Microarrays (SAM) algorithm implemented in R “samr” v1.24 to identify differentially expressed genes using an un-paired or paired approach as applicable (thus when comparing within cell lines, non-contractile vs. contractile from the same donor, a paired test was used, otherwise we used a non-paired test). We further used a fixed s_0_ = 0.1, a large delta table (400) and a fixed random seed (1,2,3,4,5,6,7,8,9) [23]. Only genes that were classified as present in at least 6 samples (in the studied comparison) using the present absent algorithm from MAS5 implemented in the “affy” package in R, were used as input as this reduced the noise [24]. We used GO::Termfinder v0.72 [25] to identify gene ontologies that were overrepresented in the generated gene lists and considered all with an False Discovery Rate (FDR)<15% significant (using simulation significances implemented in GO::Termfinder).

### Study of pathway activity

To test whether selected pathways were active in a specific comparison we sought to identify enrichment of genes within a pathway at the extreme ends of a ranked gene list. First all measured genes were ranked according to their transcription and translational “d-scores” (obtained from a SAM run including all available genes) comparison by comparison. We used a “step down” approach to test for enrichment of genes within the pathways from each end of the ranked gene list. First the range of statistics between the highest and lowest 1% d-scores was used to create 40 bins. Exclusion of the extreme 1% avoided outlier statistics from dominating the definition of the bins. For each pathway, we used Fisher's exact test to look for enrichment of genes until a given bin, compared to the total number of genes in the pathway-and the data set (thus first looking at the top 1% and then stepping down bin by bin). The analysis was performed both from the top and bottom of the ranked gene list, thus assessing activity of the pathway in the IPF and the control group. This generated matrixes of p-values and odds ratios for each pathway and from each direction. As many p-values were generated, we corrected for multiple testing using Benjamin & Hochsberg multiple correction (implemented in fdrtool for R) of all p-values, from the pathways included in the analysis. To summarize the data across all pathways and studies in a matrix, we created a discrete output so that [significant overrepresentation from the top] (1), [significant overrepresentation from the bottom] (−1), [no significant overrepresentation] (0) or [significant overrepresentation from both top and bottom] (2) was indicated in each comparison to pathway interaction. Pathways showing an FDR<0.05 were considered significant. We tested all modules at the same time, and therefore the correction for multiple testing accounted for all modules tested. This approach is similar to [26].

## Results

### Selection of time point for global analysis of transcription and ribosome recruitment

**Figure 1:**
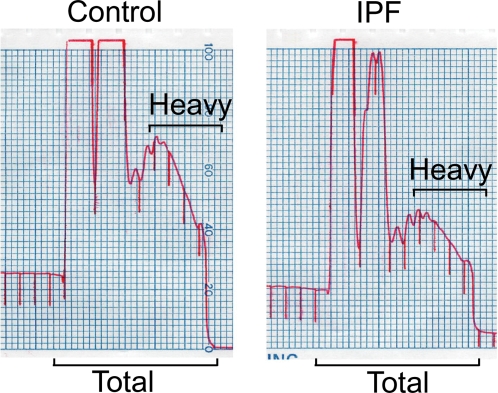
Representative polyribosome tracings from control and IPF myofibroblasts in non-contractile collagen matrices. Shown is OD 254 as a function of position in the sucrose gradient. The fractions pooled to yield the heavy polyribosomes and total RNA are designated

Three-dimensional collagen gels have been used to simulate tissue repair [27]. Because type I collagen is the most abundant matrix component in the lung interstitium, collagen gels are a useful *in vitro* model to investigate the molecular pathways regulating lung myofibroblast function. We previously showed that normal lung myofibroblasts cultured in contractile collagen gels undergo apoptosis as they contract the matrix, whereas myofibroblasts within non-contractile matrices remain viable [28], [29], [30], [31]. To ensure we were studying gene expression in viable cells, we sought a time point when differences in the intrinsic myofibroblast phenotype could be assessed independent of cell death; we used stability of ribosome loading onto RNA as the viability metric. Within 2 hours of seeding myofibroblasts into gels, cells attached and spread (observed by phase contrast microscopy–data not shown). After 4 additional hours, polyribosome tracings from IPF and control myofibroblasts in contractile and non-contractile collagen gels showed no global shift in ribosome loading (Figure 1); this time point (6 h post-seeding) was therefore selected for genome-wide assessment of transcription and ribosome recruitment to RNA.

### Genome-wide assessment of transcriptional and translational profiles identifies large changes at the translational level in IPF myofibroblasts

**Figure 2:**
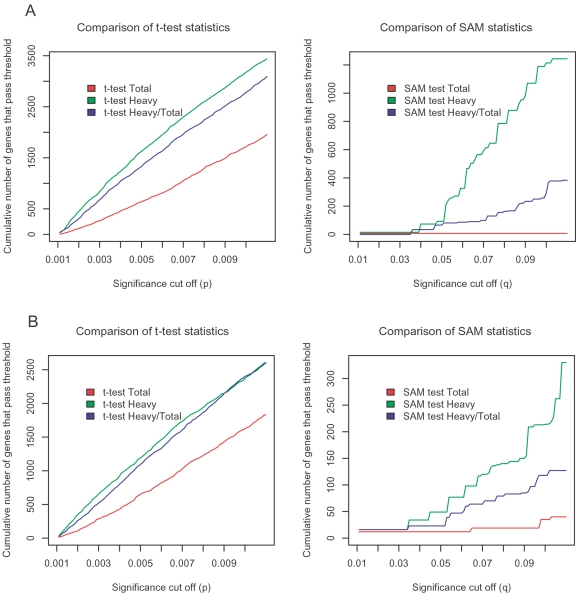
Number of genes passing significance thresholds. Shown are t-test and SAM statistics of the cumulative number of genes passing significance thresholds for transcription (red line), translation (green line), and the transcriptionally corrected translational activity (blue line). Figure 2A represents data from the non-contractile condition, and 2B shows data from the contractile collagen matrices.

We carried out a genome-wide analysis of transcript abundance and ribosome recruitment in primary lung myofibroblast lines derived from 6 patients with IPF and 6 patient controls. Transcript abundance was quantified using total RNA while assessment of ribosome recruitment was performed using RNA associated with more than 3 ribosomes (isolated using polyribosome RNA preparations as described previously [32], [33]) genome wide using microarrays. To assess whether there were intrinsic differences between IPF and control myofibroblasts, we analyzed transcript abundance and ribosome recruitment in both contractile and non-contractile collagen matrices. We characterized the extent of differential regulation of transcription (i.e. transcript abundance) and translation (i.e. ribosome recruitment) between control and IPF myofibroblasts by monitoring the cumulative number of genes passing a range of significance thresholds using both the Significance Analysis of Microarrays (SAM) algorithm and Student's t-test. We considered the possibility that the variance in the data sets generated from polyribosome RNA and total RNA might differ (so that data derived from polyribosome RNA would contain more technical noise due to increased sample processing). In this scenario SAM offers the best estimate of the magnitude of regulation since the internal data set variance directly influences the significance estimates through the sample permutation strategy built into this approach. This is in contrast to fold changes in which increased data set variance can randomly produce more extreme fold-changes. We used three different data inputs: i) data derived from total RNA, ii) data derived from the translationally active (i.e. polyribosome-associated) RNA pool and iii) translational data that had been corrected for total RNA abundance by taking the ratio of (transcript abundance in the actively translated pool)/(total transcript abundance) cell line for cell line. At each significance level, more genes differed between IPF and control at a translational than at a transcriptional level in both contractile and non-contractile gels (Figure 2). It is important to note that the transcriptionally corrected translational regulation also showed more significant regulation compared to the total RNA analysis. This finding indicates that the difference between the translational and the transcriptional regulation cannot be explained by higher data set variance for the total RNA data sets (as the fold differences in the translational estimate have been normalized to the fold differences in the transcriptional estimate and therefore contain the variance from both of these comparisons). This corrected analysis will underestimate the translational regulation as a result of the added variance from both the transcriptional and translational data. We also directly compared the data set variance and fold change distribution in these data sets (Figure S1). As expected, the polyribosome data sets showed higher variance (presumably due to the multi-step sample preparation) and also more genes with extreme fold-changes. Since differences in data set variance are controlled in the SAM algorithm, we conclude that there is substantial translational deregulation in IPF fibroblasts compared to controls that cannot be explained by transcriptional regulation.

To identify a set of differentially expressed genes that we considered significant, we used SAM and collected genes with a False Discovery Rate<15% using transcription data and data derived from the translationally active pool. Differences in transcript abundance between IPF and control were modest among the more than 15,000 measured genes. In non-contractile collagen matrices, 23 named, unique genes displayed statistically significant transcriptional differences; and in contractile collagen matrices 41 named, unique genes differed. In contrast, translation differed sharply between IPF and control. In non-contractile gels we identified 1346 named, unique genes showing significantly different ribosome recruitment, and in contractile gels there were 488 genes that differed (See Tables S1, S2, S3, S4 for full list of transcriptionally and translationally regulated genes in both contractile and non-contractile condition).

**Figure 3:**
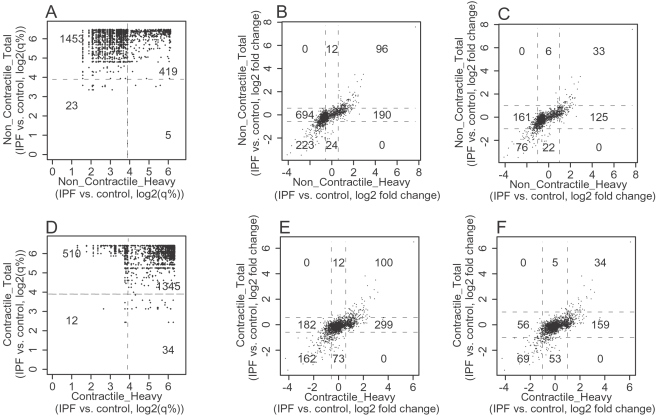
A comparison of translational and transcriptional regulation. All genes that were classified as differentially expressed (translational or transcriptional level) between IPF and control were collected. The differential regulation at the transcriptional (“Total”) or translational (“Heavy”) were compared in the non-contractile (A–C) and contractile (D–F) state. Significance level (log2 q-value (%)) (A, D) and fold changes (log2) (B–C, E–F) were used for comparison. The lines in the comparison of significances (A, D) indicate a q-value of 15% (3.9 on the log2 scale). The lines in the comparisons of fold changes indicate a 1.5 fold change (B, E) and a 2 fold change (C, F). The number of genes what fall within each sector is indicated. Higher fold changes and lower significances indicate differential regulation.

To compare the regulation patterns of genes found to be differentially expressed in any comparison (contractile, non-contractile, total RNA or polyribosomal RNA), we collected all such genes (not only those genes that were named) and compared both the significance levels and the fold changes (Figure 3, and Table S5). The analysis indicates that there is a large set of genes that are regulated at the translational level whose differential expression cannot be appreciated at the transcriptional level (see both the q-value analysis and the fold-change analysis in Figure 3) in both the contractile and the non-contractile state. In accord with the data presented in Figure 2, this fraction of genes is larger than the fraction for which regulation at the translational and transcriptional level is congruent (i.e. no translational regulation). These data demonstrate that IPF myofibroblasts differ from control primarily at the level of ribosome recruitment and that these differences are apparent in a non-contractile matrix simulating fibrosis, and persist in a contractile collagen matrix that simulates normal healing.

### Validation of differential ribosome recruitment

**Figure 4:**
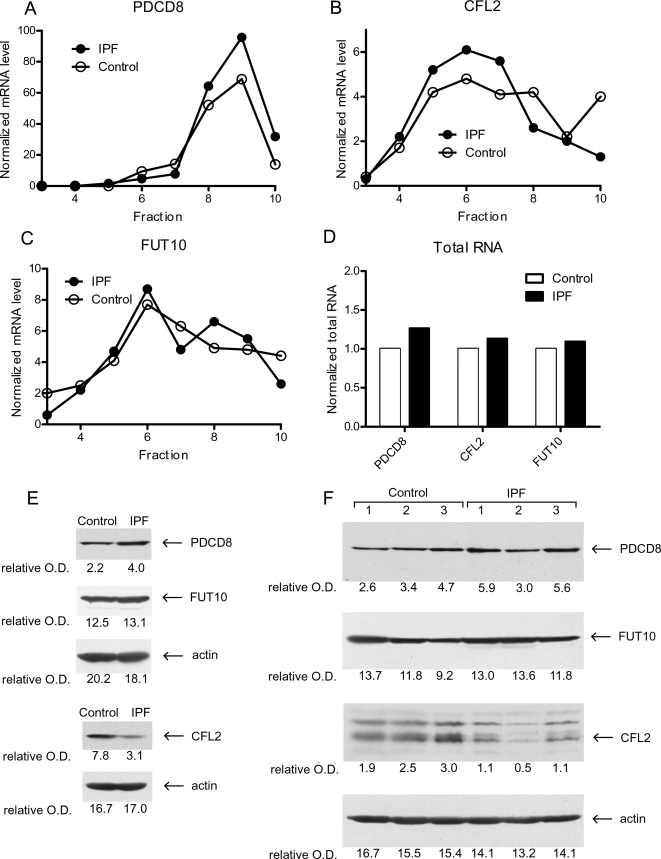
Validation of genome-wide data. Three genes were selected: Programmed Cell Death 8 (PDCD8) which demonstrated more ribosome loading in IPF; Cofilin 2 (CFL2) which showed more ribosome loading in controls; and Fucosyltransferase 10 (FUT10) which displayed no change in ribosome loading between the two cell types. A–C. Polyribosome shift using qRT-PCR. Shown is the quantity of mRNA normalized to a spike in standard, as a function of position in the sucrose gradient. D. Total RNA levels. The total RNA was normalized to a “spike in” standard and actin. E–F. Steady state protein levels of PDCD8, CFL2 and FUT10. Cells (passage 5) were seeded on type I collagen matrices and examined for protein expression using actin as a loading control E. Western blotting from the same cell lines as in (A–D). F. Steady state levels of 6 additional primary myofibroblast lines (3 IPF; and 3 control).

While the combined polyribosome-microarray approach has been used and validated in established cell lines [32], [34], [35], [36], we wanted to test its validity in primary cells. For validation of polyribosome microarray data it is common to trace the profile of the gene of interest using quantitative real time PCR (qRT-PCR) across the fractions of the polyribosome gradient. Regulation is identified as a shift towards fractions with more or fewer ribosomes depending on the direction of regulation. To do this, we identified two cell lines, one control and one IPF (new cell lines not part of the initial microarray study); that were available at a sub-cultivation number identical to that used in the microarray experiments and in sufficient quantity to generate enough mRNA for qRT-PCR from each fraction (an independent validation with new cell lines). We randomly selected one gene shown by our analysis to manifest increased ribosome recruitment in IPF (PDCD8), one gene with decreased ribosome recruitment in IPF (CFL2) and one that did not differ between IPF and control (FUT10). We performed qRT- PCR across the polyribosome fractions for these three genes in both IPF and control to assess the translational activity and measured total RNA levels to assess transcriptional regulation. For the regulated genes, the expected shift in polyribosome profile was observed (PDCD8 shifted towards higher fractions in IPF (Figure 4A); CFL2 shifted towards lower fractions in IPF (Figure 4B); and the negative control (FUT10) displayed a similar pattern in IPF and control (Figure 4C)). The total RNA level was similar for each gene in IPF compared to control (Figure 4D).

In general, increased ribosome loading is expected to lead to increased steady state levels of the encoded protein. To assess this relationship between ribosome recruitment and protein level, we performed immunoblot analysis of PDC8, CFL2 and FUT10 from the cells used for polyribosome qRT-PCR validation in Figure 4A–D. In accord with the polyribosome microarray data and the polyribosome qRT-PCR validation, immunoblot analysis showed that IPF myofibroblasts had increased PDC8 protein levels, decreased CFL2 levels and similar FUT10 levels compared to control (Figure 4E).

To expand our validation set, we identified 6 additional primary fibroblast lines (3 IPF and 3 control, which were part of the initial microarray study) that matched the sub-cultivation criteria for the original array analysis and assessed steady state protein levels for PDCD8, CFL2 and FUT10 (this requires substantially fewer cells than is needed for polyribosome preparations) (Figure 4F). For the cell lines assessed in Figure 4F, there was a mean increase in PDCD8 protein level of 1.5 fold in IPF. When also including the cell lines validated in Figure 4E, 6 out of 8 cell lines confirmed that the PDCD8 protein is more abundant in IPF. For CLF2, a similar analysis indicated a 2-fold mean difference in protein level in Figure 4F with all 8 cell lines from Figure 4E–F confirming the direction of regulation (higher in control). For FUT10 there was no mean difference (1.1 fold higher in IPF) in Figure 4F.

To assess the significance of the validation, we used the binomial distribution in which the expected pattern can either be confirmed or not between a pair of IPF and control cells (using data from 4E–F). For PDCD8 and CFL2, 7 out of 8 theoretical IPF and control pairs followed the expected pattern of regulation with 4 comparisons for CLF2 showing higher levels in control and 3 comparisons for PDCD8 showing higher levels in IPF. One pair for PDCD8 showed an opposite pattern of regulation with the control higher than IPF (note that we have used the most disadvantageous construction of pairs to obtain one failed pair). Using the binomial distribution to calculate the probability of finding 7 or more confirmed patterns of regulation out of 8 (assuming a 0.5 probability for success in each trial) , we observed a successful validation with a p-value of 0.035. If we include the additional validation of Keratin 18 (below), we observed a successful validation with a p-value of 0.006 (10 out of 11). Thus our data reflect authentic differences at the ribosome recruitment step of gene expression regulation which corresponds to changes in protein level.

### Collagen matrix state modulates translational activity

We next examined the direction of change among the translationally regulated genes. Of the 1346 genes with a significant translational shift in non-contractile collagen matrices, surprisingly only 138, or 10%, were relatively more active in IPF myofibroblasts compared with controls. However, of the 488 unique genes showing significant translational differences in contractile collagen matrices, 348, or 71%, exhibited greater ribosome loading in IPF myofibroblasts than in controls (see Table S1, S2) These data indicate that collagen matrix state modulates translational activity.

Our findings also indicate that at least one of the cell types must undergo extensive translational regulation in a matrix-dependent manner. To assess which of the cell types gave rise to this effect, we compared control and IPF myofibroblasts on both matrices. When examining translation in non-contractile vs. contractile collagen matrices, we found more than 3-fold unique, named genes differing at the level of ribosome recruitment in IPF myofibroblasts compared to control myofibroblasts (1753 *vs.* 575). Very few significant differences were found when analyzing transcriptional data in the same manner–just 26 genes differed in the IPF myofibroblasts and no genes differed in controls when comparing non-contractile vs. contractile matrices. This analysis shows that IPF cells undergo large scale translational regulation depending on matrix state, while control myofibroblasts show relative stability at this level of gene regulation.

### Systems analysis of myofibroblast function and origin

Comparisons of genes that differ between IPF and normal myofibroblasts could give important information about IPF biology. Such an assessment could be done at a single gene level or by studying the activity of groups of genes organized into pathways. When comparing the single gene approach and the “gene set” or “module” approach, modular analysis has the advantage of giving more biological information and providing a far more robust statistical environment. We therefore used both approaches to study the biology of IPF.

The gene ontology consortium[37] has developed a system for classifying biological information that has been extensively used to categorize and analyze microarray data. We used this organizational scheme to examine genes classified as differentially expressed at the translational level (too few genes were identified at the transcriptional level to provide a meaningful analysis). One comparison (more active in controls in contractile matrices) resulted in no significant functions (FDR<15% was used as a significance threshold). Two other comparisons (more active in IPF in contractile matrices and more active in IPF in non-contractile matrices) resulted in several significant functions. These included membrane and vesicle transport functions in the non-contractile state and membrane and metabolism (primarily carbohydrate, protein and glycoprotein synthesis) in the contractile state (Table S6, S7). In the final analysis of genes that were more active in control compared to IPF myofibroblasts in the non-contractile state, we found a striking enrichment of genes involved in cell cycle regulation (Table S8). These genes included both positive and negative cell cycle regulators, indicating that translational control of cell cycle regulation differs between IPF and control; a result in accord with the current literature [38], [39], [40], [41]. Thus this functional analysis indicated that depending on matrix state, different functions differentiated IPF from controls at the translational level.

One possible explanation for the differences observed regarding cell cycle regulation is that we are studying myofibroblast populations or subpopulations that have begun to enter cellular senescence. This concern emerged from our observation that IPF myofibroblasts showed morphological changes characteristic of senescence [42] at an earlier passage than did controls (data not shown). While our experiments employed cells several passages before any morphological senescence was observed, we wanted to investigate whether differences between IPF and controls in our analysis could be attributable to IPF cells just beginning to enter senescence. We therefore compared our microarray data to a data set describing a meta-signature from senescent cells apparent across many cell types and species [43]; we found no indication for senescence in our primary cell lines.

As another explanation of the differences between IPF and control myofibroblasts, we considered the possibility that a small subpopulation of rapidly proliferating cells could dominate the results. To address this issue, we applied a technique commonly used in immunology to track the proliferation of subpopulations of lymphocytes and labeled our myofibroblasts with the stable vital dye, carboxyfluoroscein succinimidyl ester (CFSE) [44]. Uniform proliferation on a population level can be observed as Gaussian distribution whose mean CFSE signal is decreased by approximately 50% per cell division. Using this procedure, we determined that cell proliferation was uniform in all IPF and control cell lines, with an approximate doubling time of 1 day. There was neither a rapidly nor a slowly proliferating subpopulation in any of the cell lines studied (Figure S2).

The conventional systems approach we employed to analyze our gene lists has important limitations that arise from its dependence on finite lists of individual genes that pass an arbitrary significance threshold. Such lists are dominated by well-expressed genes experiencing large changes. These lists do not take into account biologically important genes that may barely-or even fail to-pass a significance or fold-change threshold, but nevertheless are critical components of a physiological process that my be the crux of the pathobiology under study [45]. It is therefore important to supplement these conventional analyses with methods that are not dependent on finite lists of significant genes.

**Figure 5:**
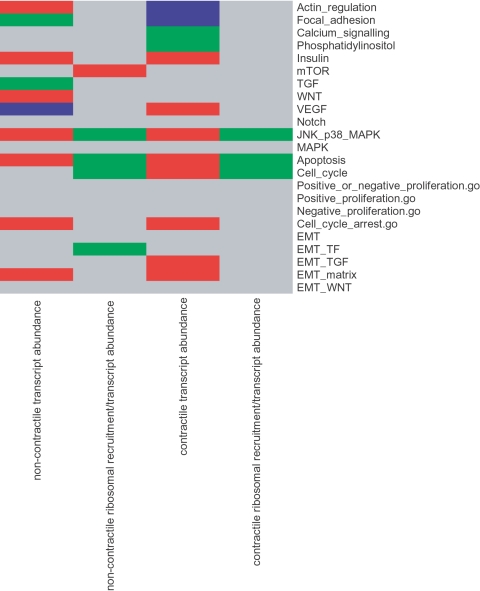
Systems analysis of pathway activities and myofibroblast origin. Pathways are shown in rows and conditions are shown in columns. A red box denotes that the pathway is activated in IPF compared with control myofibroblasts; green boxes represent pathways that are activated in controls compared to IPF myofibroblasts. Blue boxes signify a pathway that is activated both in IPF compared to controls and controls compared to IPF, and gray boxes represent pathways with no significant activity. This analysis was performed using cells in non-contractile matrices setting the threshold at a FDR<0.05, (“TF” = transcription factors). Pathways ending with “.go” were obtained from GO. EMT pathways were manually constructed while all other pathways were obtained from KEGG.

We therefore used a modular approach that has proved useful in prior studies of translational control [45]. All genes were ordered based on relative transcriptional or translational activity in IPF compared to control myofibroblasts, as described in Materials and Methods. We looked for over-representation of genes belonging to selected pathways at the extreme ends of the ordered list, thus assessing increased or decreased activity of the pathway in IPF compared to control myofibroblasts. It should be noted that these pathways are different than the GO-collection studied above except for 4 modules as indicated in Figure 5; and that the significance levels were corrected for multiple testing taking into account all analyses presented in Figure 5. We selected all published pathways available for cell signaling (found on the web at http://www.genome.ad.jp/kegg), and detected significant differences in pathway activities at the level of transcript abundance and ribosome recruitment (Figure 5). Some pathways, such as transforming growth factor (TGF) and actin regulation, have established roles in fibrotic processes. Others, such as wingless (Wnt) or calcium signaling, are less commonly associated with fibrosis but may lead to novel insights into the disease process. We also observed regulation of apoptosis and proliferation at both the transcriptional and translational level. The observed differential regulation of proliferation motivated us to further examine cell cycle regulation using data from additional sources and we collected several categories from GO that describe proliferation. However, only the “cell cycle arrest” pathway was more active in IPF compared to normal myofibroblasts. Thus by using curated pathways and genome-wide data, we find support for a pathological myofibroblast phenotype in IPF that persists *in vitro* up to 9 sub-cultivations. It is noteworthy that this phenotype is manifest at both levels of gene regulation examined as well as in several biological pathways.

One critical question amenable to a systems level approach is the origin of the IPF myofibroblast. The epithelial to mesenchymal transition (EMT) is a key process during embryonic development that has been implicated as a source of pathological myofibroblasts in renal fibrosis and IPF [46], [47], [48]. To examine whether there was genome-wide indication for EMT at the level of transcription and/or translation, we manually constructed a module comprised of 111 genes that had at least one published study documenting its participation in EMT, and built 4 sub-modules to represent the 3 major cell surface receptor-mediated pathways triggering EMT (Wnt, TGF-β and integrin-matrix) and a module comprised of EMT-related transcription factors (module gene lists provided in Table S9; these modules were not assessed in the “significant gene list analysis” presented above). The global EMT module was not active in IPF compared to control (Figure 5). When studying the subgroups the TGF-β -EMT module, the integrin-matrix-EMT module and the transcription factor module distinguished IPF from control–primarily at the level of transcript abundance. The analysis shown in Figure 5 was corrected for multiple testing, and thus the corrected significance level (q<0.05) considers all tests performed in this part of our study. As a result, p<0.001 was necessary to reach a corrected significance level of q<0.05. These data support the contention that EMT is involved in the genesis of some IPF myofibroblasts, and show the power of this systems level analysis.

**Figure 6:**
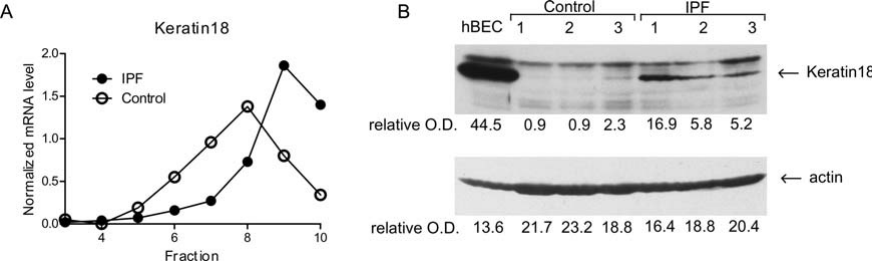
Keratin 18 is translationally activated in IPF myofibroblasts. A. Polyribosome shift using qRT-PCR. Shown is the quantity of mRNA normalized to a “spike in” standard, as a function of position in the sucrose gradient. B. Steady state protein level. Cells (passage 5) were seeded on type I collagen matrices and examined for protein expression using actin as a loading control.

To test the biological validity of this systems level analysis implicating EMT in the origin of IPF myofibroblasts, we selected the epithelial intermediate filament component, Keratin 18, for further analysis. In our genome-wide analysis of ribosome recruitment, we found that this epithelial gene was dramatically translationally activated in IPF myofibroblasts compared to controls. To verify this we used the same samples and approach as in Figure 4A–C to assess the level of Keratin 18 mRNA across the polyribosome fractions. Figure 6A shows that Keratin 18 was indeed more translationally active in IPF compared to control. Keratin 18 was also marginally increased at the total RNA level (<1.5 fold, not shown). Nonetheless, to rule out transcriptional regulation as a primary source of the translational difference, we corrected the translational profiling data for total RNA levels. This did not influence the shift towards translational activation in IPF (not shown). To assess Keratin 18 expression in a panel of cell lines, we used the same samples as in Figure 4F (i.e. independent samples from 3 IPF and 3 control fibroblast lines which were part of the microarray study) and measured protein abundance by immunoblot (mammary epithelial cells served as a positive control, Figure 6B). In accord with the genome-wide study and the polyribosome qRT-PCR validation, Keratin 18 displayed an increase in protein abundance (Figure 6B) in IPF myofibroblasts compared to controls. These data indicate that our approach is biologically valid, and lends further support for the idea that EMT is involved in the origin of IPF fibroblasts.

## Discussion

Myofibroblasts from fibrotic lesions manifest pathological control of proliferation, viability, motility, contractile function and connective tissue production. In a prior report, we provided the first insights into molecular mechanism, showing that aberrant beta 1 integrin signaling results in defective PTEN function, unrestrained Akt signaling and downstream activation of the translation initiation machinery [16]. Here, we provide the first genome-wide analysis of the consequences of this aberrant signaling. We find that two steps in the flow of genetic information–transcription and ribosome recruitment-are altered in IPF myofibroblasts, and that changes in ribosome recruitment account for the majority of differences between IPF and control myofibroblasts. When comparing gene expression in contractile and non-contractile matrices, IPF myofibroblast gene expression showed large-scale translational changes depending on matrix state, whereas the pattern in control myofibroblasts was relatively stable. In addition, our analysis provides systems level evidence for EMT as a source of some IPF myofibroblasts, providing strong support for the pathological study suggesting an epithelial origin for some IPF myofibroblasts [47]. Our data do not exclude the possibility that other IPF myofibroblasts may originate from cytokine altered resident fibroblasts or from circulating fibrocytes. These findings do, however, establish the power of systems level genome-wide analysis to provide mechanistic insights into IPF, and point to derangements of translational control downstream of defective integrin signaling as a fundamental component of IPF pathobiology.

The clinical outcome of IPF is the result of complex interactions among myofibroblasts, epithelial cells, cytokines, and the surrounding extracellular matrix. Significant debate continues as to what degree, or even whether, an abnormality in any individual component contributes to the overall disease process. Part of this uncertainty relates to conflicting results found in studies of IPF myofibroblasts. As an example, investigators comparing proliferation and apoptosis between IPF and control myofibroblasts have reached contradictory conclusions[38], [39], [40], [41]. While many aspects of the debate are not resolved by the results of this study, there is no doubt that these key processes are fundamentally different in IPF and control myofibroblasts.

Our study demonstrates this fundamental difference in a comprehensive microarray analysis of two levels in the gene expression pathway–transcription and ribosome recruitment. A few previous studies have been performed using microarray technology to compare transcriptional profiles of IPF and control myofibroblasts [49], [50]. Microarray analysis has also been done using whole lung samples from patients with IPF, hypersensitivity pneumonitis (HP), nonspecific interstitial pneumonitis (NSIP) and controls [17], [51], [52]. However, all of these investigations focus on differences in mRNA abundance, which correlates poorly with protein levels [53], [54]. Analysis of translation, the next step in the processing of genetic information, reveals more prominent and informative differences between cells, and correlates more closely with protein levels [32], [34]. Consistent with these data, our study demonstrates markedly more genes differing between IPF and control myofibroblasts at the level of ribosome recruitment compared with mRNA abundance. Our study is also the first to perform this analysis using primary cells rather than immortal, established cell lines.

Analysis of the stability of the transcriptional and translational profiles between IPF and control as a function of matrix state revealed major differences. Control myofibroblasts demonstrated no significant differences in transcript abundance and relatively few differences in ribosome recruitment on non-contractile compared to contractile matrices. In contrast, IPF myofibroblasts displayed extensive changes in transcription and ribosome recruitment as matrix state changed. This analysis illustrates a difference in phenotype between IPF and control myofibroblasts, suggesting that in IPF there is pathologic relaxation of the gene expression control system found in normal cells. Furthermore, these data fit with the idea that IPF myofibroblast pathobiology includes a loss of translational control, analogous to the loss of tumor suppressor function seen in cancer. Experimental precedent for this concept has been provided by studies showing that IPF myofibroblasts have acquired at least one cancer-related property–the ability to grow in an anchorage-independent manner [55].

Given the growing body of experimental work indicating that fibrotic myofibroblasts have a distinct phenotype, one topic that has recently garnered much interest is the source of myofibroblasts in IPF. Published morphological data suggest that myofibroblasts in IPF have an epithelial origin [56]; and there is direct experimental data implicating EMT as a source of myofibroblasts in a mouse model of lung fibrosis [47]. Using a systems approach to analyze genome-wide data, we establish that myofibroblasts cultured from the lungs of patients with IPF have an EMT signature. At least 3 pathways can trigger EMT. Here we group genes associated with each of the 3 EMT pathways, and find that two of the pathways (matrix and TGF) are active in IPF myofibroblasts–providing the first systems-level indication regarding mechanism. In accord with this result, recent data indicates that myofibroblast contraction of its extracellular matrix can trigger release of TGF- β from its latent form in the matrix [57].

In this report, we demonstrate a difference in transcript abundance and ribosome recruitment for a number of genes when comparing myofibroblasts from patients with IPF to controls. We chose a systems biology approach rather than a “reductionist” or “cherry picking” method to analyze our data [45], [58]. We show instability in the translational profile of IPF myofibroblasts when they are placed in different matrix environments, and we present genome-wide data that provide indications for EMT as a source of myofibroblasts in IPF. We anticipate these data, along with more intensive investigations of primary cell lines, will yield important information and significantly impact the search for new molecular targets for therapeutics.

### Accession number

The data set has been deposited at Gene Expression Omnibus (GEO, GSE11196).

## Supporting Information

Figure S1 (0.96 MB EPS)

Figure S2 (0.94 MB EPS)

Table S1 (0.21 MB XLS)

Table S2 (0.45 MB XLS)

Table S3 (0.05 MB XLS)

Table S4 (0.05 MB XLS)

Table S5 (0.52 MB XLS)

Table S6 (0.03 MB XLS)

Table S7 (0.04 MB XLS)

Table S8 (0.05 MB XLS)

Table S9 (0.05 MB XLS)
